# A Dangerous Dentist

**Published:** 1876-05

**Authors:** 


*A Dangerous Dentist.*—The following ridiculous story is told by the Paris *Gaulois* of a certain dentist who has just been consigned to the Mazas prison in Paris on the charge of poisoning certain of his patients. He is stated to have induced them to pay him frequent visits, and under pretense of attending to their defective teeth, to have introduced small doses of poison into their hollow molars. These specially favored individuals are supposed to have been sent to him by their heirs or by other persons interested in their removal. More than two-hundred witnesses are stated to be in readiness. Inquiries of the police authorities have shown that the first wife of the prisoner (who is now married a second time,) died suddenly under very suspicious circumstances, and that two women with whom he had disreputable relations also disappeared mysteriously. The first wife of the prisoner was possessed of a considerable fortune, of which he came into exclusive possession at her death. It is expected that the trial will disclose some extraordinary revelations.

